# Identification of a Novel Angiogenesis Signalling circSCRG1/miR-1268b/NR4A1 Pathway in Atherosclerosis and the Regulatory Effects of TMP-PF In Vitro

**DOI:** 10.3390/molecules28031271

**Published:** 2023-01-28

**Authors:** Rong Yuan, Qiqi Xin, Xiaochang Ma, Meng Yu, Yu Miao, Keji Chen, Weihong Cong

**Affiliations:** 1 Cardiovascular Diseases Laboratory, Xiyuan Hospital, China Academy of Chinese Medical Sciences, Beijing 100091, China; 2 National Clinical Research Center for Chinese Medicine Cardiology, Xiyuan Hospital, China Academy of Chinese Medical Sciences, Beijing 100091, China

**Keywords:** atherosclerosis, plaque stability, cardio-cerebrovascular disease, neovascularization, circRNA, Chinese medicine, tetramethylpyrazine, paeoniflorin

## Abstract

Angiogenesis contributes to plaque instability in atherosclerosis and further increases cardio-cerebrovascular risk. Circular RNAs (circRNAs) are promising biomarkers and potential therapeutic targets for atherosclerosis. Previous studies have demonstrated that tetramethylpyrazine (TMP) and paeoniflorin (PF) combination treatment (TMP-PF) inhibited oxidized low-density lipoprotein (ox-LDL)-induced angiogenesis in vitro. However, whether circRNAs regulate angiogenesis in atherosclerosis and whether TMP-PF can regulate angiogenesis-related target circRNAs in atherosclerosis are unknown. In this study, human RNA sequencing (RNA-seq) data were analysed to identify differentially expressed (DE) circRNAs in atherosclerosis and to obtain angiogenesis-associated circRNA-microRNA (miRNA)-messenger RNA (mRNA) networks. Target circRNA-related mechanisms in angiogenesis in atherosclerosis and the regulatory effects of TMP-PF on target circRNA signalling were studied in ox-LDL-induced human umbilical vein endothelial cells (HUVECs) by cell proliferation, migration, tube formation, and luciferase reporter assays, real-time quantitative polymerase chain reaction (RT-qPCR) and Western blotting. A novel circRNA (circular stimulator of chondrogenesis 1, circSCRG1) was initially identified associated with angiogenesis in atherosclerosis, and circSCRG1 silencing up-regulated miR-1268b expression, increased nuclear receptor subfamily 4 group A member 1 (NR4A1) expression and then promoted ox-LDL-induced angiogenesis. TMP-PF (1 μmol/L TMP combined with 10 μmol/L PF) up-regulated circSCRG1 expression, mediated miR-1268b to suppress NR4A1 expression and then inhibited ox-LDL-induced angiogenesis. However, circSCRG1 silencing abolished the inhibitory effects of TMP-PF on ox-LDL-induced angiogenesis, which were rescued by the miR-1268b inhibitor. In conclusion, circSCRG1 might serve as a new target regulating angiogenesis in atherosclerosis via the circSCRG1/miR-1268b/NR4A1 axis and TMP-PF could regulate the circSCRG1/miR-1268b/NR4A1 axis to inhibit angiogenesis in atherosclerosis in vitro, indicating a novel angiogenesis signalling circSCRG1/miR-1268b/NR4A1 pathway in atherosclerosis and the regulatory effects of TMP-PF, which might provide a new pharmaceutical strategy to combat atherosclerotic plaque instability.

## 1. Introduction

Atherosclerotic plaque rupture, the main cause of most cardio-cerebrovascular events, is a major global health problem. Thus, plaque stabilization is beneficial for reducing the risk of acute cardio-cerebrovascular events [1]. Plaque angiogenesis plays a crucial role in the plaque microenvironment, often associated with plaque destabilization and causes the majority of acute cardiovascular events [2]. Patients with plaque angiogenesis generally feature high plaque volumes and plaque burden; thus, plaque angiogenesis can predict the risk of plaque progression [3]. The selective depletion of plaque angiogenesis by therapeutic ultrasound can improve vulnerable plaque stability [4]. In the atherosclerotic pathological process, oxidized low-density lipoproteins (ox-LDLs) tend to activate the vascular endothelial growth factor (VEGF) pathway, leading to endothelial cell proliferation, cell migration, vascular tube formation and increased angiogenesis, thus inducing plaque instability [2,5]. Plaque neovessels and intraplaque haemorrhages have been reported to colocalize with VEGF/VEGF receptor 2 (VEGFR2), while VEGFR2 blocking resulted in a significant 44% decrease in intraplaque haemorrhages [1,6]. Therefore, certain angiogenesis-related factors might be potential therapeutic targets for plaque instability, and elucidation of the underlying molecular mechanisms is of great clinical importance.

Circular RNAs (circRNAs), a new type of endogenous noncoding RNAs that regulate the transcriptional and post-transcriptional levels of genes, are regarded as potential biomarkers and therapeutic targets for cardiovascular disease [7,8,9,10]. circRNAs exert essential biological functions by sponging specific microRNAs (miRNAs), binding RNA-binding proteins, translating proteins, interfering with signalling pathways, and modulating transcription and splicing [11,12,13,14]. Construction of circRNA-miRNA-messenger RNA (mRNA) networks might help identify new potential diagnostic and therapeutic targets for diseases; for example, circ_0002722-miR-130-PPARG might be associated with the pathogenesis of abdominal aortic aneurysm; this finding thus provides potential therapeutic targets for abdominal aortic aneurysm [15]. A large number of studies have shown that circRNAs are involved in the occurrence and development of atherosclerosis and may be considered as targets for clinical treatment [16]. However, whether circRNAs can regulate angiogenesis and serve as new targets for plaque instability is not yet known.

At present, the therapeutic effects of anti-angiogenic drugs are mediated via the delay of plaque progression and increased plaque stability by targeting the plaque microvasculature [17]. However, clinical studies on anti-angiogenic therapies for atherosclerosis or cardio-cerebrovascular diseases are not available. In addition, some anti-angiogenic drugs tend to aggravate the pathological changes that occur in myocardial ischaemia, heart failure, hypertension, stroke, thromboembolic diseases, and thrombotic microangiopathy [18], reflective of the side effects of single-target therapies [19]. Thus, drugs that inhibit angiogenesis in atherosclerotic plaques without cardiovascular side effects may provide new therapeutic options for atherosclerosis and cardio-cerebrovascular diseases.

According to traditional Chinese medicine (TCM) theory, atherosclerosis-related diseases are usually treated by activating blood circulation and removing blood stasis (ABCRS). The Chuanxiong-Chishao herbal pair (Chuanxiong Rhizoma and Paeoniae Radix Rubra) is a commonly used combination in some of the most famous ancient ABCRS formulas. It has a long history in the prevention and treatment of atherosclerosis and cardio-cerebrovascular diseases in China, as it has demonstrated significant protective effects against atherosclerosis, endothelial dysfunction, myocardial ischaemia and cerebral ischaemia according to the results of clinical and basic research [20,21]. The material basis of the effects of TCM is believed to mainly come from a formula’s active ingredients, which has been proven by numerous studies, including those on the prevention and treatment of atherosclerosis [22]. Tetramethylpyrazine (TMP, C_8_H_12_N_2_, molecular weight: 136.22, oral bioavailability: 20.01%, soluble in hot water, petroleum ether, chloroform and dilute hydrochloric acid) is the main active alkaloid separated and purified from Chuanxiong Rhizoma *(Ligusticum chuanxiong* Hort.), which exhibits antioxidant, antiapoptotic and anti-inflammatory properties and regulates autophagy, angiogenesis, vasodilation, endothelial function and so on [23]. Paeoniflorin (PF, C_23_H_28_O_11_, molecular weight: 480.51, oral bioavailability: 53.87%, soluble in water, methanol, ethanol and chloroform) is the main active ingredient of Paeoniae Radix Rubra (*Paeonia lactiflora* Pall.), which exerts anti-inflammatory, antioxidant, antithrombotic, antitumoral, antidepressant, cardioprotective, hepatoprotective and neuroprotective effects and regulates immunity, angiogenesis and so on [24]. Previous work has demonstrated that TMP and PF used in combination (TMP-PF) suppresses angiogenesis in ox-LDL-induced endothelial cells, an in vitro atherosclerosis model, by blocking the VEGF/VEGFR2 and Jagged1/Notch1 signalling pathway, and TMP-PF was more effective than TMP or PF alone [25]. However, whether TMP-PF inhibits angiogenesis in atherosclerosis by regulating angiogenesis-related target circRNAs is not yet clear.

This study aimed to identify differentially expressed (DE) circRNAs in atherosclerosis using human RNA sequencing (RNA-seq) data, construct angiogenesis-associated target circRNA-miRNA-mRNA networks by bioinformatics analysis, and subsequently find a target circRNA-miRNA-mRNA network that regulates angiogenesis in atherosclerosis. This study also aimed to observe the regulatory effects of TMP-PF on target circRNA signalling in ox-LDL-induced angiogenesis in vitro.

## 2. Results

### 2.1. CircRNA Screening and circRNA-miRNA-mRNA Network Establishment

**Figure 1:**
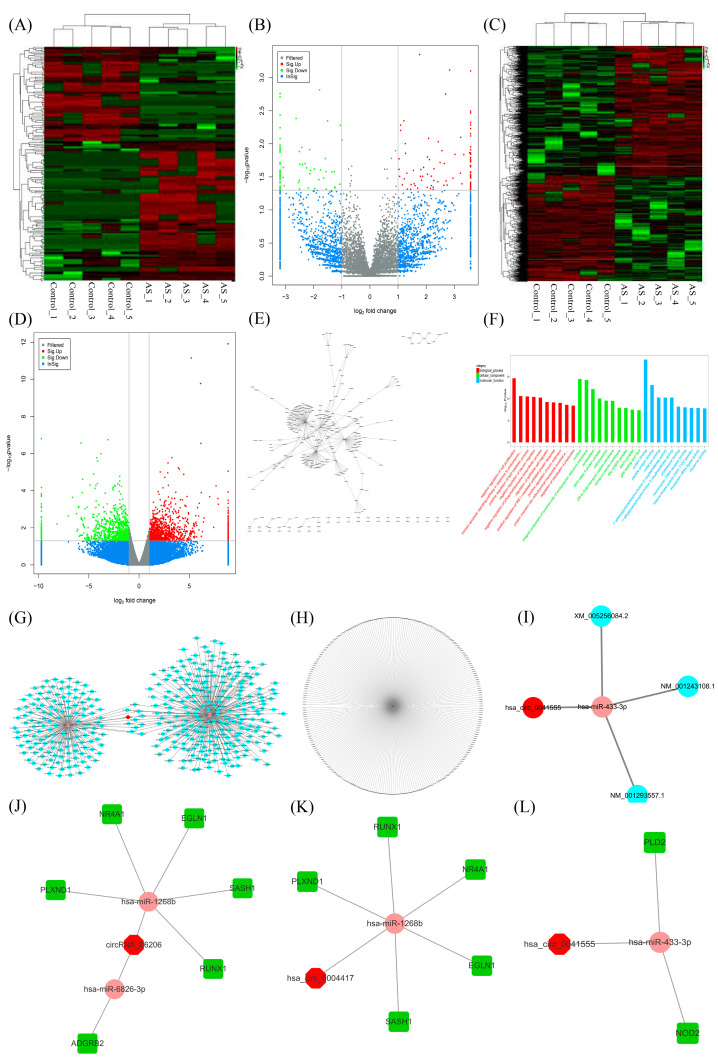
Identification of differentially expressed (DE) circular RNAs (circRNAs), DE messenger RNAs (mRNAs) and circRNA–microRNA (miRNA)–mRNA networks based on RNA sequencing data analysis of samples from atherosclerotic patients. (**A**) Heatmap of the DE circRNAs between atherosclerotic patients (AS) and healthy volunteers (control). Red indicates high intensity, green indicates low intensity, and black indicates medium intensity. (**B**) Volcano plot of DE circRNAs; green dots represent low DE circRNAs, and red dots represent high DE circRNAs with statistical significance. (**C**,**D**) Heatmap and volcano plot of the DE mRNAs between atherosclerotic patients (AS) and healthy volunteers (control). (**E**) The overlapping miRNAs predicted as downstream targets of the DE circRNAs, and the top 300 circRNA-miRNA networks were established. (**F**) Gene ontology (GO) functional enrichment analysis of DE mRNAs. (**G**–**I**) The overlapping miRNAs predicted as downstream targets of circRNA_06206, hsa_circ_0004417, and hsa_circ_0041555 and upstream regulators of the DE mRNAs were enriched, and then circRNA_06206−, hsa_circ_0004417−, and hsa_circ_0041555−involved circRNA-miRNA-mRNA networks associated with atherosclerosis were established. (**J**–**L**) The overlapping miRNAs predicted as downstream targets of circRNA_06206, hsa_circ_0004417, and hsa_circ_0041555 and upstream regulators of the DE mRNAs associated with angiogenesis were enriched, and then circRNA_06206−, hsa_circ_0004417−, and hsa_circ_0041555−involved circRNA-miRNA-mRNA networks that regulate both atherosclerosis and angiogenesis were established. circRNA-miRNA-mRNA networks were delineated using Cytoscape 3.6.0. Red circular nodes represent circRNAs, pink circular nodes represent miRNAs, blue circular nodes represent mRNAs, and green square nodes represent mRNAs associated with angiogenesis.

To further study the in-depth mechanisms involving circRNAs in angiogenesis in atherosclerosis, RNA-seq data of plasma samples from atherosclerotic patients and healthy volunteers were analysed to screen angiogenesis-associated target circRNAs and their network partners. DE circRNAs and genes between atherosclerotic patients and healthy volunteers were screened, and 202 DE circRNAs and 2179 DE mRNAs were obtained. Heatmaps and volcano plots of the DE circRNAs (Figure 1A,B) and DE mRNAs (Figure 1C,D) were used to visualize the data. Then, networks consisting of the top 300 DE circRNAs and miRNAs (circRNA-miRNA networks) were established (Figure 1E). Moreover, gene ontology (GO) enrichment analysis of the DE mRNAs revealed the enrichment of cell proliferation, migration and protein binding (Figure 1F). Of the 202 DE circRNAs and 2179 DE mRNAs in atherosclerosis, three novel DE circRNAs were selected, namely, circRNA_06206, hsa_circ_0004417 and hsa_circ_0041555, and the relevant circRNA-miRNA-mRNA networks were constructed (Figure 1G–I). Then, angiogenesis-related mRNAs were screened, and three DE circRNA-miRNA-mRNA networks associated with both atherosclerosis and angiogenesis were constructed (Figure 1J–L).

### 2.2. Verification of a Target circRNA and Its Role in ox-LDL-Induced Angiogenesis

**Figure 2:**
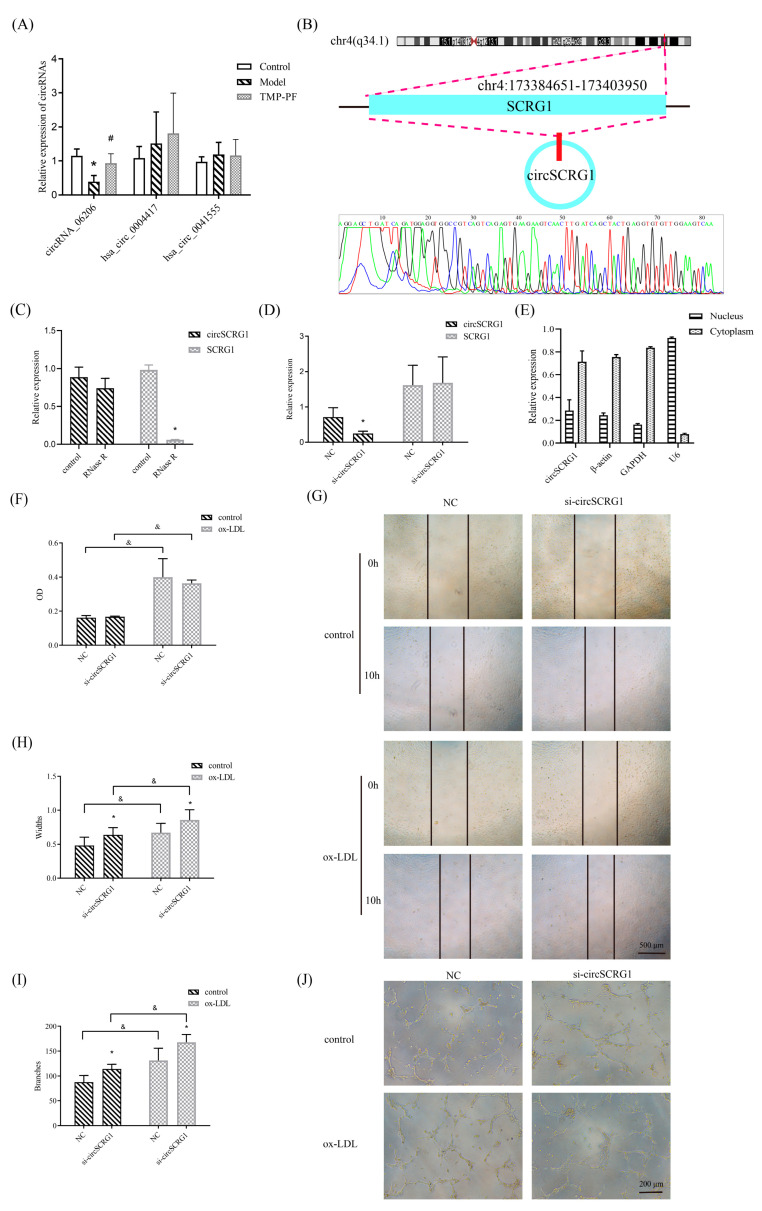
Verification of a target circRNA and its anti-angiogenic effects in oxidized low-density lipoprotein (ox-LDL)-induced human umbilical vein endothelial cells (HUVECs). (**A**) HUVECs were induced with 20 μg/mL ox-LDLs for 24 h to establish a model and then treated with TMP-PF (1 μmol/L TMP combined with 10 μmol/L PF) for 24 h. The relative expression levels of circRNA_06206, hsa_circ_0004417, and hsa_circ_0041555 in HUVECs were evaluated by real-time quantitative polymerase chain reaction (RT-qPCR). (**B**) Spliced mature sequences of circular stimulator of chondrogenesis 1 (circSCRG1/circRNA_06206) derived from stimulator of chondrogenesis 1 (SCRG1). (**C**) Cells were incubated with or without RNase R and the relative expression levels of circSCRG1 and SCRG1 were evaluated by RT-qPCR. (**D**) The relative expression of circSCRG1 and SCRG1 in HUVECs after 100 nmol/L siRNA targeting circSCRG1 (si-circSCRG1) intervention. (**E**) The cellular localization of circSCRG1 was detected by a nuclear–cytoplasmic separation assay, and the expression of circSCRG1 in the nucleus and cytoplasm of HUVECs was evaluated by RT-qPCR. U6 was used as a positive control in the nucleus, and β-actin and glyceraldehyde-3-phosphate dehydrogenase (GAPDH) were used as positive controls in the cytoplasm. (**F**) Control or ox-LDL-induced HUVECs were transfected with 100 nmol/L si-circSCRG1 or negative control (NC) for 24 h. Cell proliferation was measured by a methyl thiazolyl tetrazolium (MTT) assay and expressed as the optical density (OD). (**G**) Representative photomicrographs showing the migration of control or ox-LDL-induced HUVECs transfected with 100 nmol/L si-circSCRG1 or NC for 24 h (scale bar: 500 μm). (**H**) Quantification of width, which represents cell migration, in wound healing assays. (**I**) Quantification of branches, which represent tube formation, in Matrigel tube formation assays. (**J**) Representative photomicrographs showing the tube formation of control or ox-LDL-induced HUVECs transfected with 100 nmol/L si-circSCRG1 or NC for 24 h (scale bar: 200 μm). One-way analysis of variance (ANOVA) with the LSD post hoc test was used to compare multiple groups, and Student’s *t* test was used to compare two groups. Data are the mean ± standard deviations (SDs), * *p* < 0.05 versus the NC group, # *p* < 0.05 versus the model group, & *p* < 0.05 versus the corresponding control group.

To further determine the roles of circRNAs in regulating ox-LDL-induced angiogenesis, three DE circRNAs associated with both atherosclerosis and angiogenesis were screened in ox-LDL-induced human umbilical vein endothelial cells (HUVECs). The expression of circRNA_06206 was suppressed after ox-LDL induction, whereas TMP-PF promoted the expression of circRNA_06206 (Figure 2A), suggesting that circRNA_06206 participates in the regulation of angiogenesis in atherosclerosis. Therefore, circRNA_06206 was initially identified as a target circRNA and named the circular stimulator of chondrogenesis 1 (circSCRG1) in this study since it was generated from its host gene, stimulator of chondrogenesis 1 (SCRG1) (Figure 2B). This novel circRNA was submitted to the GenBank database under accession number MK732524. It was confirmed that circSCRG1 was resistant to RNase R, while linear SCRG1 mRNA was degraded (Figure 2C), and small interfering RNA (siRNA) targeting circSCRG1 (si-circSCRG1) disrupted the expression of circSCRG1 but had no effect on SCRG1 linear mRNA levels (Figure 2D), all of which confirmed the circularization and stability of circSCRG1. Furthermore, real-time quantitative polymerase chain reaction (RT-qPCR) of nuclear and cytoplasmic fractions showed that circSCRG1 was mainly localized in the cytoplasm (Figure 2E). Then, the angiogenic functions of circSCRG1 in normal and ox-LDL-induced HUVECs were examined. The results of the methyl thiazolyl tetrazolium (MTT) assay showed that ox-LDLs promoted cell proliferation compared with the corresponding control group (Figure 2F). The wound healing assay showed that ox-LDLs promoted cell migration compared with the corresponding control group, and si-circSCRG1 also promoted cell migration compared with the negative control (NC) group in normal and ox-LDL-induced HUVECs (Figure 2G,H). The Matrigel tube formation assay showed that ox-LDLs promoted cell tube formation compared with the corresponding control group, and si-circSCRG1 also promoted cell tube formation compared with the NC group in normal and ox-LDL-induced HUVECs (Figure 2I,J), indicating that the effects of si-circSCRG1 were similar to the proangiogenic effects of ox-LDLs. These results showed that circSCRG1 silencing induced angiogenesis, suggesting that circSCRG1 is an important circRNA that regulates angiogenesis in atherosclerosis.

### 2.3. The circSCRG1/miR-1268b/Nuclear Receptor Subfamily 4 Group A Member 1 (NR4A1) Signalling-Regulated Angiogenesis in Atherosclerosis

**Figure 3:**
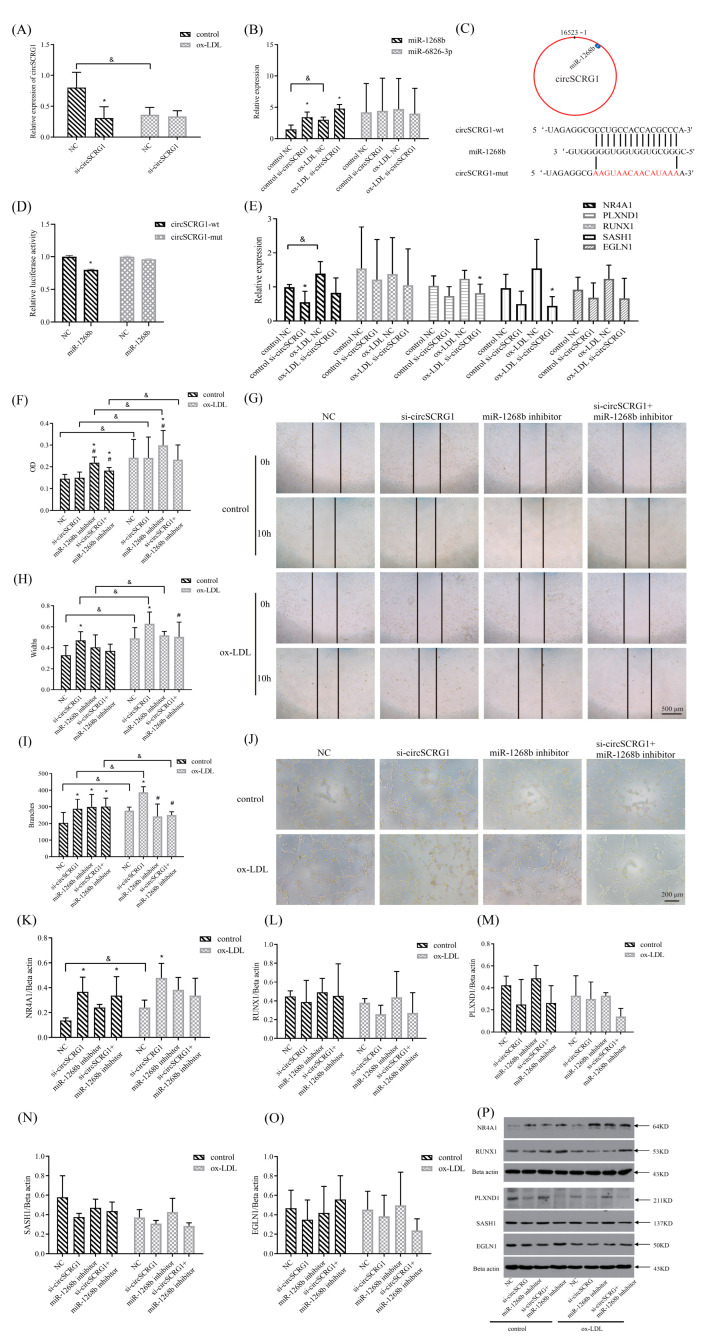
si-circSCRG1 promoted ox-LDL-induced angiogenesis by interacting with miR-1268b to up-regulate the target gene nuclear receptor subfamily 4 group A member 1 (NR4A1). (**A**,**B**) Control or ox-LDL-induced HUVECs were transfected with 100 nmol/L si-circSCRG1 or NC for 24 h. The relative expression levels of circSCRG1, miR-1268b and miR-6826-3p were evaluated by RT-qPCR. (**C**) Schematic of putative miR-1268b-binding sites on circSCRG1 and sequence alignment of circSCRG1 with miR-1268b analysed with the miRanda, RNAhybrid and TargetScan databases. (**D**) Luciferase activities of wild-type circSCRG1 (circSCRG1-wt) or mutant circSCRG1 (circSCRG1-mut) following transfection with an miR-1268b mimic or NC. (**E**) Control or ox-LDL-induced HUVECs were transfected with 100 nmol/L si-circSCRG1 or NC for 24 h. The relative expression levels of egl nine homologue 1 (EGLN1), NR4A1, plexin D1 (PLXND1), runt-related transcription factor 1 (RUNX1) and SAM and SH3 domain-containing 1 (SASH1) were evaluated by RT-qPCR. (**F**) Control or ox-LDL-induced HUVECs were transfected with 100 nmol/L NC, si-circSCRG1, and/or an miR-1268b inhibitor for 24 h. Cell proliferation was measured by an MTT assay and expressed as the OD value. (**G**) Representative photomicrographs showing the migration of control or ox-LDL-induced HUVECs transfected with 100 nmol/L NC, si-circSCRG1, and/or an miR-1268b inhibitor for 24 h (scale bar: 500 μm). (**H**) Quantification of width, which represents cell migration, in wound healing assays. (**I**) Quantification of branch points, which represent tube formation, in Matrigel tube formation assays. (**J**) Representative photomicrographs showing the tube formation of control or ox-LDL-induced HUVECs transfected with 100 nmol/L NC, si-circSCRG1, and/or an miR-1268b inhibitor for 24 h (scale bar: 200 μm). (**K**–**O**) Control or ox-LDL-induced HUVECs were transfected with 100 nmol/L NC, si-circSCRG1, and/or an miR-1268b inhibitor for 24 h. The relative expression levels of the NR4A1, RUNX1, PLXND1, SASH1 and EGLN1 proteins were evaluated by Western blotting. (**P**) Representative Western blot images of NR4A1, RUNX1, PLXND1, SASH1 and EGLN1. Student’s *t* test was used to compare two groups, and one-way ANOVA with the LSD post hoc test was used to compare multiple groups. Data are mean ± SDs, * *p* < 0.05 versus the NC group, # *p* < 0.05 versus the si-circSCRG1 group, & *p* < 0.05 versus the corresponding control group.

To investigate circSCRG1 signalling in ox-LDL-induced angiogenesis, the relationships between circSCRG1, its predicted target miRNAs (miR-1268b and miR-6826-3p) and target genes were further studied. The results showed that both ox-LDLs and si-circSCRG1 down-regulated circSCRG1 expression and up-regulated miR-1268b expression, but neither affected miR-6826-3p expression (Figure 3A,B). Thus, the target miRNA (miR-1268b) was confirmed, and the sequences of circSCRG1 and miR-1268b were used to conduct dual-luciferase reporter assays (Figure 3C). The luciferase activity of wild-type circSCRG1 (circSCRG1-wt) cells was significantly reduced by miR-1268b, while that of mutant circSCRG1 (circSCRG1-mut) cells was unaffected, suggesting that circSCRG1 interacts with miR-1268b (Figure 3D). Then, target genes of miR-1268b, such as NR4A1, egl nine homologue 1 (EGLN1), SAMand SH3 domain-containing 1 (SASH1), runt-related transcription factor 1 (RUNX1), and plexin D1 (PLXND1), were further studied after circSCRG1 silencing in normal and ox-LDL-induced HUVECs. Ox-LDLs up-regulated NR4A1 gene expression, while si-circSCRG1 inhibited NR4A1 gene expression in normal HUVECs; si-circSCRG1 also inhibited the expression of the PLXND1 and SASH1 genes in ox-LDL-induced HUVECs, while the expression of other genes did not significantly change (Figure 3E).

Subsequently, the angiogenic functions of circSCRG1 and miR-1268b in normal and ox-LDL-induced HUVECs were examined. In normal HUVECs, si-circSCRG1 promoted cell migration and tube formation compared with the NC group, and the miR-1268b inhibitor alone or in combination with si-circSCRG1 significantly promoted cell proliferation and tube formation compared with the NC group (Figure 3F–J). In ox-LDL-induced HUVECs, si-circSCRG1 promoted cell migration and tube formation compared with the NC group, whereas the miR-1268b inhibitor alone or in combination with si-circSCRG1 significantly inhibited tube formation compared with the si-circSCRG1 group (Figure 3H,I). These results indicated that circSCRG1 silencing aggravated ox-LDL-induced angiogenesis, while the miR-1268b inhibitor nullified the ability of si-circSCRG1 to promote angiogenesis in ox-LDL-induced HUVECs, suggesting that circSCRG1 inhibited angiogenesis by interacting with miR-1268b in the ox-LDL-induced HUVECs. Then, the protein levels of the target genes in normal and ox-LDL-induced HUVECs were observed. The protein expression of NR4A1 was increased after ox-LDL induction (Figure 3K). In normal HUVECs, si-circSCRG1 increased NR4A1 protein expression, while the miR-1268b inhibitor did not affect the si-circSCRG1-mediated increase in NR4A1 protein expression (Figure 3K). In ox-LDL-induced HUVECs, si-circSCRG1 increased NR4A1 protein expression, which was blunted by the miR-1268b inhibitor (Figure 3K). There was no significant change in the expression of other proteins (Figure 3L–P). These results indicated that si-circSCRG1 might promote angiogenesis by interacting with miR-1268b to up-regulate NR4A1 expression in ox-LDL-induced HUVECs, indicating that the circSCRG1/miR-1268b/NR4A1 axis might regulate angiogenesis in atherosclerosis.

### 2.4. TMP-PF Suppressed ox-LDL-Induced Angiogenesis by Regulating the circSCRG1/miR-1268b/NR4A1 Axis

**Figure 4:**
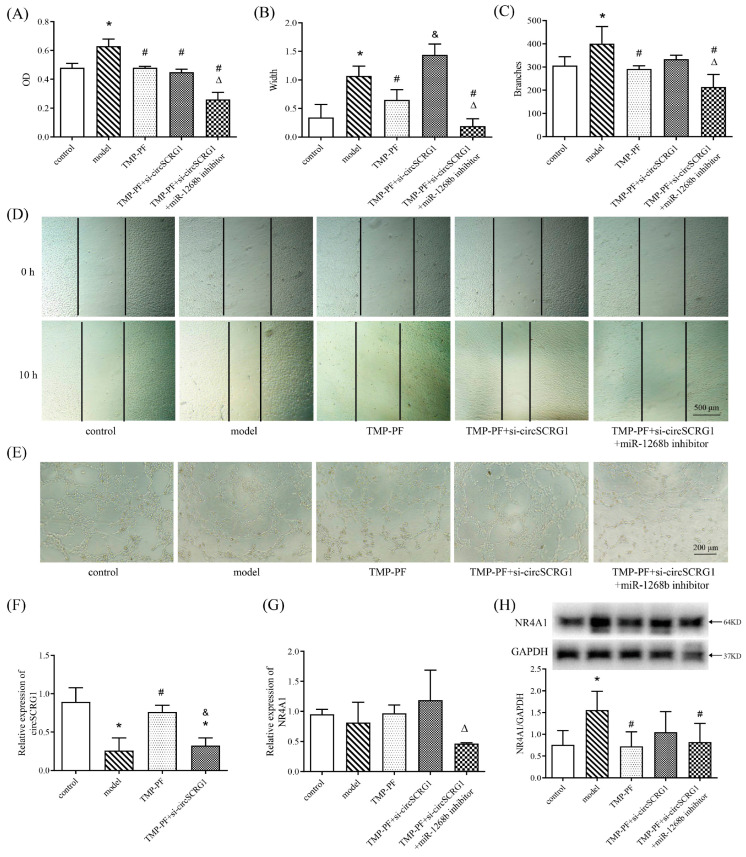
TMP-PF suppressed ox-LDL-induced angiogenesis by regulating the circSCRG1/miR-1268b/NR4A1 axis. (**A**) HUVECs were transfected with 100 nmol/L NC, miR-1268b inhibitor and/or si-circSCRG1 for 24 h, after which 20 μg/mL ox-LDLs were added for 24 h to establish the model, and then the HUVECs were treated with TMP-PF (1 μmol/L TMP combined with 10 μmol/L PF) for 24 h. Cell proliferation was measured by an MTT assay and expressed as the OD value. (**B**) Quantification of width, which represents cell migration, in wound healing assays. (**C**) Quantification of branch points, which represent tube formation, in Matrigel tube formation assays. (**D**) Representative photomicrographs showing the migration of ox-LDL-induced HUVECs transfected with 100 nmol/L NC, si-circSCRG1, and/or an miR-1268b inhibitor for 24 h and treated with TMP-PF (1 μmol/L TMP combined with 10 μmol/L PF) for 24 h (scale bar: 500 μm). (**E**) Representative photomicrographs showing the tube formation of ox-LDL-induced HUVECs transfected with 100 nmol/L NC, si-circSCRG1, and/or an miR-1268b inhibitor for 24 h and treated with TMP-PF (1 μmol/L TMP combined with 10 μmol/L PF) for 24 h (scale bar: 200 μm). (**F**,**G**) HUVECs were transfected with 100 nmol/L NC, miR-1268b inhibitor and/or si-circSCRG1 for 24 h, after which 20 μg/mL ox-LDLs were added for 24 h to establish the model, and then the HUVECs were treated with TMP-PF (1 μmol/L TMP combined with 10 μmol/L PF) for 24 h. The relative expression levels of circSCRG1 and NR4A1 were evaluated by RT-qPCR. (**H**) The relative protein expression of NR4A1 was evaluated by Western blotting. One-way ANOVA with the LSD post hoc test was used to compare multiple groups. * *p* < 0.05 versus the control group, # *p* < 0.05 versus the model group, & *p* < 0.05 versus the TMP-PF group, Δ *p* < 0.05 versus the TMP-PF+si-circSCRG1 group.

To investigate whether TMP-PF could regulate circSCRG1/miR-1268b/NR4A1 signalling to inhibit angiogenesis in atherosclerosis, ox-LDL-induced HUVECs undergoing angiogenesis were treated with TMP-PF, si-circSCRG1 and an miR-1268b inhibitor. The results of the MTT, wound healing and Matrigel tube formation assays showed that cell proliferation, cell migration and tube formation were increased in the model group compared with the control group, whereas TMP-PF suppressed ox-LDL-induced cell proliferation, cell migration and tube formation compared with the model group (Figure 4A–E), indicating that TMP-PF suppressed ox-LDL-induced angiogenesis. However, the TMP-PF+si-circSCRG1 group showed increased cell migration compared with the TMP-PF group, and the TMP-PF+si-circSCRG1+miR-1268b inhibitor group showed decreased cell migration compared with the TMP-PF+si-circSCRG1 group (Figure 4B), indicating that si-circSCRG1 reversed the effects of TMP-PF on ox-LDL-induced cell migration, while the miR-1268b inhibitor rescued these effects. In addition, si-circSCRG1 did not abrogate the repressive effects of TMP-PF on ox-LDL-induced cell proliferation but abolished its repressive effects on cell tube formation, and the miR-1268b inhibitor blocked the effects of si-circSCRG1 and rescued the inhibitory effects of TMP-PF on ox-LDL-induced cell proliferation and tube formation (Figure 4A,C), all of which suggested that TMP-PF inhibited ox-LDL-induced angiogenesis via the regulation of circSCRG1/miR-1268b. Moreover, ox-LDL inhibited circSCRG1 expression and promoted NR4A1 protein expression compared with the control group, and TMP-PF promoted circSCRG1 expression and suppressed NR4A1 protein expression compared with the model group, but there was no difference in NR4A1 mRNA expression (Figure 4F–H). However, si-circSCRG1 eliminated the effects of TMP-PF on the expression of circSCRG1 and the NR4A1 protein, and the miR-1268b inhibitor nullified the effects of si-circSCRG1 and rescued the inhibitory effect of TMP-PF on NR4A1 protein expression (Figure 4F,H). Taken together, these findings suggest that TMP-PF might inhibit ox-LDL-induced angiogenesis through the circSCRG1/miR-1268b/NR4A1 axis.

## 3. Discussion

**Figure 5:**
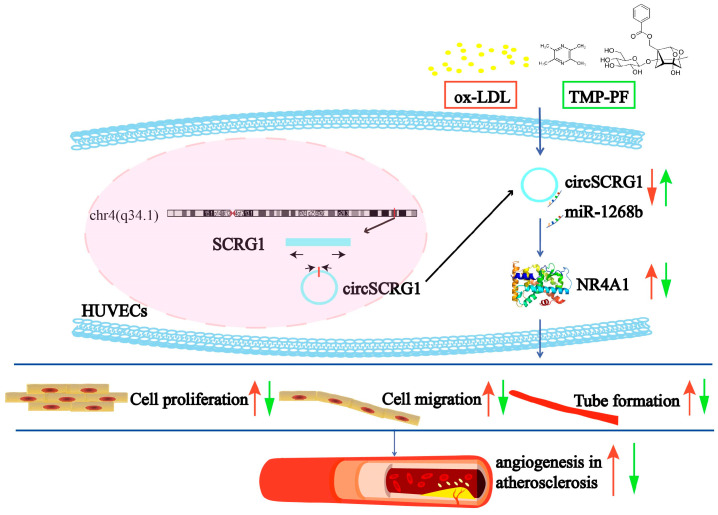
Schematic diagram showing the mechanisms of circSCRG1-mediated angiogenesis in atherosclerosis and the inhibitory effects of TMP-PF. Red arrows indicate the effects after ox-LDL induction, and green arrows indicate the effects after TMP-PF treatment.

In this study, we first identified DE circRNAs and circRNA-miRNA-mRNA networks associated with angiogenesis in atherosclerosis, after which we verified a novel circSCRG1 and provided evidence, for the first time, that the circSCRG1/miR-1268b/NR4A1 axis regulates ox-LDL-induced angiogenesis in vitro. We also preliminarily confirmed that TMP-PF regulates the circSCRG1/miR-1268b/NR4A1 axis to inhibit ox-LDL-induced angiogenesis in vitro and ultimately exerts anti-atherosclerotic effects (Figure 5). The results of this study confirm circSCRG1 as a new angiogenesis target for plaque instability and circSCRG1/miR-1268b/NR4A1 as a novel angiogenesis signalling pathway in atherosclerosis. This study also suggests that TMP-PF can inhibit angiogenesis in atherosclerosis by regulating circSCRG1 signalling. Taken together, these results provide a novel pharmaceutical strategy to combat atherosclerotic plaque instability.

Recent studies have shown that circRNAs have important biological functions in atherosclerosis. For example, the circMAP3K5/miR-22-3p/TET2 axis might participate in the treatment of intimal hyperplasia-related diseases, including atherosclerosis [26]; circARHGAP12 ameliorated atherosclerosis progression through the miR-630/EZH2/TIMP2 axis [27]; melatonin inhibited aortic valve calcification through the circRIC3/miR-204-5p/DPP4 pathway in valvular interstitial cells [28]; and the circRNA-0006896/miR-1264/DNMT1 axis played a crucial role in plaque stabilization [29]. However, few studies have reported the antiangiogenetic effects of circRNAs in atherosclerosis. The present study provides the first evidence that circSCRG1 was significantly down-regulated in ox-LDL-induced HUVECs, and circSCRG1 silencing induced angiogenesis and up-regulated NR4A1 expression, effects that were blunted by an miR-1268b inhibitor, indicating that the circSCRG1/miR-1268b/NR4A1 axis might be closely associated with angiogenesis in atherosclerosis, suggesting a novel angiogenesis signalling pathway in atherosclerosis. Since most circRNAs are abundant in bodily fluids and highly stable, making them ideal candidates for diagnostic biomarkers [8,9], the abnormal expression of circSCRG1 is suggested to act as a potential prognostic biomarker for plaque instability.

Some studies have indicated that certain circRNAs have miRNA-binding sites and inhibit miRNA activity by acting as miRNA sponges [30,31], while other studies have suggested that most circRNAs do not function as miRNA sponges [32,33]. Interestingly, in this study, circSCRG1 silencing increased NR4A1 expression by interacting with miR-1268b in ox-LDL-induced HUVECs but directly regulated NR4A1 in normal HUVECs, which indicated that the circSCRG1/miR-1268b/NR4A1 axis might be more closely associated with a pathological state than a normal state. In other words, the correlation between circSCRG1 and miR-1268b might be more positive in atherosclerosis than under healthy conditions. It is worth noting that miR-1268b partially conveys the function of circSCRG1, which might represent a new regulatory mechanism that differs from the sponge mechanism [34]. Furthermore, since a single circRNA can regulate multiple miRNAs and a single gene is modulated by multiple miRNAs [35], understanding whether circSCRG1 has other target miRNAs and whether NR4A1 is regulated by other miRNAs in atherosclerotic angiogenesis requires further investigation.

The characteristics of multiple TCM components limit the elucidation of their pharmacological effects and underlying mechanisms. The present study on TMP-PF has partially clarified the mechanism by which the Chuanxiong-Chishao herbal pair affects angiogenesis in atherosclerosis, which might provide a way to investigate the mechanism of complex ABCRS formulas in TCM. Previous clinical and experimental evidence has demonstrated that the Chuanxiong–Chishao herbal pair and its active constituents (Xiongshao capsule) have beneficial effects on restenosis after percutaneous coronary intervention, atherosclerosis and ischemic heart disease, and the mechanisms were found to be related to antiplatelet aggregation, anti-inflammatory, and the improvement of endothelial function and myocardial ischaemia [20,36]. The results of this study initially revealed that TMP-PF suppressed ox-LDL-induced angiogenesis and NR4A1 expression and promoted circSCRG1 expression, while circSCRG1 silencing abolished the repressive effects of TMP-PF on angiogenesis and NR4A1 expression in ox-LDL-induced HUVECs. In addition, the miR-1268b inhibitor blocked the effects of circSCRG1 silencing and rescued the inhibitory effects of TMP-PF on ox-LDL-induced angiogenesis and NR4A1 expression. These results demonstrated for the first time that TMP-PF ameliorated ox-LDL-induced angiogenesis through the circSCRG1/miR-1268b/NR4A1 axis in vitro, indicating a new pharmaceutical strategy to combat atherosclerotic plaque instability.

In the present study, NR4A1 was found to be the main target gene of circSCRG1 and the therapeutic target of TMP-PF in the regulation of angiogenesis in atherosclerosis. A previous study showed that NR4A1 was a critical transcription factor in VEGF pathway-mediated angiogenesis, and NR4A1 silencing could significantly inhibit VEGF-induced angiogenesis [37]. Previous studies have primarily focused on the bidirectional roles of NR4A1 in the inflammatory response of atherosclerosis [38] or NR4A1-mediated pro-angiogenic effects in tumour growth and skin wound healing [39,40]. However, few studies have focused on the role of NR4A1 in atherosclerotic angiogenesis. In this study, NR4A1 was highly expressed in ox-LDL-induced HUVECs, and TMP-PF down-regulated NR4A1 expression and inhibited angiogenesis, effects that were abolished by circSCRG1 silencing. Therefore, TMP-PF might promote the inhibitory effects of circSCRG1 on NR4A1, indicating that circSCRG1-mediated NR4A1 regulation could be considered as a potential therapeutic approach to combat plaque instability. Furthermore, the mRNA and protein levels of NR4A1 were inconsistent, which indicates that the regulatory effects of genes on proteins are complicated. NR4A1 gene expression might be affected by the degradation of transcription products, translation or modification, and eukaryotic transcription is spatially and temporally separated from translation [41]. Therefore, mRNA degradation when the protein levels peaked may have occurred, leading to inconsistent transcription and translation levels.

The present study has some limitations: circSCRG1 gain-of-function experiments and pull-down assays could not be performed since the sequence of circSCRG1 (16,523 bp) is too long to construct an overexpression vector with the current technology. In addition, the expression and function of circSCRG1 in pathological angiogenesis could not be examined in mice, rats or rabbits because circSCRG1 is not conserved between species. Additionally, the effects of TMP-PF on the circSCRG1/miR-1268b/NR4A1 axis in vivo could not be performed.

## 4. Materials and Methods

### 4.1. RNA-Seq Data Analysis

As reported by a previous clinical trial, RNA-seq was carried out on plasma samples from atherosclerotic patients and healthy volunteers [42], and the raw RNA-seq data were deposited in the National Center of Biotechnology Information (NCBI) Sequence Read Archive (SRA) database under the accession numbers SRR13170648–SRR13170658. In this study, the RNA-seq data from the SRA database were directly analysed by bioinformatics, and DE circRNAs and DE mRNAs between patients with atherosclerosis and healthy volunteers were compared and visualized using heatmaps and volcano plots. The functions of the DE circRNAs were evaluated by establishing circRNA-miRNA networks, and the functions of the DE mRNAs were assessed by GO analyses.

### 4.2. CircRNA-miRNA-mRNA Network Construction Based on Bioinformatic Analysis

Bioinformatic analysis was performed to screen the DE circRNAs and DE mRNAs with cut-offs of *p* ≤ 0.05 and |FC| ≥ 2. circRNA-miRNA and miRNA–mRNA interactions were predicted with the miRanda, RNAhybrid and TargetScan databases. The overlapping miRNAs predicted as downstream targets of the DE circRNAs and upstream regulators of the DE mRNAs were enriched, and then the DE circRNA-miRNA-mRNA networks involved in atherosclerosis were established as described in a previous study [43]. Then, angiogenesis-related mRNAs were used to establish atherosclerosis-related circRNA-miRNA-mRNA networks that regulate angiogenesis, and candidate circRNAs were preliminarily identified. Visual analysis was carried out with Cytoscape 3.6.0, which was used to obtain the network map.

### 4.3. Cell Culture and Treatment

HUVECs were extracted from human umbilical veins as described previously [44]. HUVECs were cultured in endothelial cell medium (1001, ScienCell, CA, USA) with 10% foetal bovine serum, 1% endothelial cell growth supplement, 0.1 mg/mL streptomycin and 100 IU/mL penicillin in a 37 °C incubator with 5% CO_2_. An in vitro atherosclerotic angiogenesis model was established with ox-LDL-induced HUVECs as indicated previously [45]. Cells were induced with 20 μg/mL ox-LDLs (H7950, Solarbio Life Science, Beijing, China) for 24 h to establish the model and then treated with TMP-PF (1 μmol/L TMP combined with 10 μmol/L PF) according to a previous study [25]. Hydrochloride TMP and PF were purchased from Shanghai Yuanye Bio-Technology Co., Ltd. (Shanghai, China). TMP and PF were dissolved in dimethyl sulfoxide, and the control group was treated with the same amount of dimethyl sulfoxide. Each in vitro experiment was repeated at least three times.

### 4.4. Target circRNA Screening

Candidate circRNAs associated with atherosclerosis were further screened. Cells (1 × 10^6^ cells/mL) were cultured in 6-well plates. After ox-LDL induction for 24 h and TMP-PF intervention for 24 h, 1 mL of TRIzol was added to each well to extract the RNA. The expression levels of candidate circRNAs were detected using RT-qPCR; from these candidates, one circRNA was selected as a target circRNA. Then, the main target circRNA-miRNA-mRNA network associated with both atherosclerosis and angiogenesis was used for the subsequent interference study.

### 4.5. Cell Transfection

The si-circSCRG1, an miR-1268b inhibitor and a corresponding NC were synthesized (RiboBio, Guangzhou, China). The sequences are listed in Supplementary Table S1. Cells were transfected with 100 nmol/L NC, siRNA, or inhibitor using the riboFECT CP transfection reagent (RiboBio, Guangzhou, China) according to the manufacturer’s instructions.

### 4.6. Cell Proliferation Assay

Cells (5 × 10^4^ cells/mL) were seeded in 96-well plates. After various experimental treatments were applied to the cells (transfection with siRNA or inhibitor for 24 h, ox-LDL induction for 24 h, and TMP-PF intervention for 24 h), 10 μL of MTT was added to each well, and the samples were then incubated at 37 °C for another 4 h. The OD was measured with a microplate reader (BioTek, Shoreline, WA, USA).

### 4.7. Cell Migration Assay

Cells (2 × 10^5^ cells/mL) were seeded in 48-well plates, and cell migration was assessed by a wound healing assay as indicated previously [46]. After cell transfection with siRNA or inhibitor for 24 h, a wound was created by applying a 200 μL pipette tip to the cell monolayer. Then, ox-LDLs were added to the cells with or without TMP-PF. Photographs were taken before and 10 h after wound generation. Migration was quantified as the difference between the initial and final width of the wound areas. The width was quantitated using ImageJ software (version 1.49).

### 4.8. Matrigel Tube Formation Assay

Tube formation was detected using the Matrigel tube formation assay as described previously [46]. Matrigel (356231, BD Biosciences, CA, USA) was added to 96-well plates and incubated for 30 min at 37 °C to allow the Matrigel to solidify. After cell transfection with siRNA or inhibitor for 24 h, ox-LDLs were added to cells with or without TMP-PF, and cell suspensions (1 × 10^5^ cells/mL) were added to the 96-well plates precoated with Matrigel basement membrane matrix. After incubation in a 37 °C incubator with 5% CO_2_ for 8 h, the gels were observed using an inverted microscope (LEICA, Wetzlar, Germany), and tube formation was quantified using ImageJ software (version 1.49). The branch points were counted in each image.

### 4.9. Luciferase Reporter Assay

A luciferase reporter assay was performed as described previously [46]. The miR-1268b-binding site of circSCRG1 was predicted with the TargetScan, miRanda and RNAhybrid databases. Cells were co-transfected with the circSCRG1-wt vector or circSCRG1-mut vector (GeneChem, Shanghai, China) and with the miR-1268b mimic or NC using X-tremeGene HP (Roche, Basel, Switzerland). After transfection for 48 h, a luciferase assay was performed using a dual-luciferase system (Promega, WI, USA). The ratio of luciferase activity was calculated. The sequences of circSCRG1-wt, circSCRG1-mut and miR-1268b mimic are listed in Supplementary Table S1.

### 4.10. RT-qPCR

After various experimental treatments were applied to the cells (transfection with siRNA or inhibitor for 24 h, ox-LDL induction for 24 h, and TMP-PF intervention for 24 h), total RNA was extracted using TRIzol reagent (Invitrogen, CA, USA) according to the manufacturer’s instructions. cDNA was synthesized and used to carry out RT-qPCR using a ViiA 7 real-time PCR system (Applied Biosystems, CA, USA). The RT-qPCR amplification parameters were as follows: 95 °C for 10 min, followed by 40 cycles of 95 °C for 10 s and 60 °C for 60 s. The relative gene expression was measured by the 2^−ΔΔCt^ method. The sequences of all primers used are listed in Supplementary Table S2.

### 4.11. Western Blotting

After various experimental treatments were applied to the cells (transfection with siRNA or inhibitor for 24 h, ox-LDL induction for 24 h, and TMP-PF intervention for 24 h), the cells were harvested and lysed, and the protein contents were measured. Cell lysates were boiled and loaded onto sodium dodecylsulfate polyacrylamide gel electrophoresis (SDS-PAGE) gels. The membranes were blocked and incubated with specific primary antibodies against NR4A1 (1:1000, Ab109180, Abcam, Cambridge, UK), PLXND1/PLXD1 (1:1000, YN2290, Immunoway, Suzhou, China), SASH1 (1:500, Ab110776, Abcam, Cambridge, UK), RUNX1 (1:1000, Ab35962, Abcam, Cambridge, UK), EGLN1/PHD2 (1:1000, 4835s, CST, Boston, MA, USA), beta actin (1:1000, TA-09, ZSGB-BIO, Beijing, China) and GAPDH (1:1000, 5174, CST, Boston, MA, USA) overnight at 4 °C. Following incubation with secondary antibody IgG (H+L) horseradish peroxidase (1:10,000, 111-035-003, Jackson, West Grove, PA, USA), the protein blots were visualized. Greyscale analysis was performed with Gel Image System 4.00 (Tanon, Shanghai, China), and relative expression was calculated.

### 4.12. Statistical Analysis

Statistical analysis was carried out using SPSS 17.0 software (SPSS, Chicago, IL, USA), and graphs and diagrams were generated using GraphPad Prism 6.07 software (GraphPad, San Diego, CA, USA). Data were expressed as the mean ± SDs. Comparisons between two groups were performed using Student’s *t* test, and comparisons among multiple groups were performed using one-way analysis of variance (ANOVA) with the LSD post hoc test. Differences for which *p* ≤ 0.05 were considered statistically significant.

## 5. Conclusions

CircSCRG1 might be a novel angiogenesis target for plaque instability, and the circSCRG1/miR-1268b/NR4A1 axis might regulate angiogenesis in atherosclerosis and mediate the anti-angiogenesis effects of TMP-PF in atherosclerosis in vitro, indicating a novel angiogenesis signalling pathway in atherosclerosis and the regulatory effects of TMP-PF. This study sheds new light on a therapeutic strategy to combat atherosclerotic plaque instability, while the function of circSCRG1 and the effects of TMP-PF on circSCRG1 signalling should be further studied in atherosclerotic patients, so as to provide more reliable evidence for the pathogenesis and treatment of atherosclerosis.

## Data Availability

All data that support the findings of this study are available from the corresponding author upon reasonable request.

## Supplementary Materials

The following supporting information can be downloaded at: https://www.mdpi.com/article/10.3390/molecules28031271/s1, Table S1: Sequences of si-circSCRG1, circSCRG1-wt, circSCRG1-mut, the miR-1268b inhibitor and the miR-1268b mimic. Table S2: Targets, primer sequences, annealing temperatures, and product lengths used in RT-qPCR.
