# Supplementary material for: Identification of a Novel Angiogenesis Signalling circSCRG1/miR-1268b/NR4A1 Pathway in Atherosclerosis and the Regulatory Effects of TMP-PF In Vitro
Source: Molecules. 2023 Jan 28;28(3):1271. doi: 10.3390/molecules28031271 (PMC9919304; doi:10.3390/molecules28031271)
Supplement: Supplementary file 1 [file molecules-28-01271-s001.zip › molecules-2089128-supplementary.pdf]

Supplementary Table S1: Sequences of si-circSCRG1, circSCRG1-wt, circSCRG1-mut, the miR-1268b inhibitor and the miR-1268b mimic

Product name	Sequence
si-circSCRG1	GCATCTGGATCAGTACCTA
circSCRG1(miR-1268b)-wt	TATATACACTTTTTTTTTGGGGGGACGGAGTCTCACTCTGTCGCCCAGGCTGGAGTGC AGTGGCACGATCTCAGCTCACTGCAAGCTCCACCTCCTGGGTTCACGCCATTCTCCTG CCTCAGCCTCCCAAATAGCTGGGACTAGAGGCGCCTGCCACCACGCCCAGCTAATTT TTTTTTTTTTTTTTTTTGTATTTTTAGTAGAGACGAGGTTTCACCGTATTAGCCAGGATG GTCTTGATCTCCTGACCTCGTGATAAGCCCGACATTTTTTATTCTAGGCAAGAGCCTA TAGAGGTCTTATGTTGTCTAAAAGAATATATGGACTTCTTGGGCTAACCTAAGGCCTT CCTTTCACTTTGTCTCCATGCCTATCATTTATTCCTGTTCAGCTAGCCGGGCAGTCAAG CCAGTCATCTCTTTAATCCTATGTATGTGTCTCTGCCTTGTTGTTCCTTCTCACCCCTTC CCTT
circSCRG1(miR-1268b)-mut	TATATACACTTTTTTTTTGGGGGGACGGAGTCTCACTCTGTCGCCCAGGCTGGAGTGC AGTGGCACGATCTCAGCTCACTGCAAGCTCCACCTCCTGGGTGACATAAATTCTCCT GCCTCAGCCTCCCAAATAGCTGGGACTAGAGGCGAAGTAACAACATAAAAGCTAAT TTTTTTTTTTTTTTTTTTTGTATTTTTAGTAGAGACGAGGTTTCACCGTATTAGCCAGGA TGGTCTTGATCTCCTGACCTCGTGATAAGCCCGACATTTTTTATTCTAGGCAAGAGCC TATAGAGGTCTTATGTTGTCTAAAAGAATATATGGACTTCTTGGGCTAACCTAAGGCC TTCCTTTCACTTTGTCTAACGTAAGATCATTTATTCCTGTTCAGCTAGCCGGGCAGTCA AGCCAGTCATCTCTTTAATCCTATGTATGTGTCTCTGCCTTGTTGTTCCTTCTCACCCCT TCCCTT
miR-1268b inhibitor	CACCCCCACCACCACGCCCG
miR-1268b mimic (sense strand)	UUUGUACUACACAAAAGUACUG

Notes: Abbreviation: si-circSCRG1, small interfering RNA (siRNA) of circular stimulator of chondrogenesis 1 (circSCRG1); circSCRG1-wt, circSCRG1-wild-type; circSCRG1-mut, circSCRG1-mutant-type.

Supplementary Table S2: Targets, primers sequence, annealing temperature, and product length used in RT-qPCR

Gene	Bidirectional primer sequence	Annealing temperature (°C)	Product length (bp)
circRNA_06206 (circSCRG1)	F:5′ GCTGTTTCACAGACACAAGCATC3′ R:5′ CTTCATCGTAATTCGCAGAGACT3′	60	86
hsa_circ_0004417	F:5′ TAGAACCACACAAGGAGAAGCC 3′ R:5′ CTGTCAAACCATACATTGGAGATT 3′	60	94
hsa_circ_0041555	F:5′ TGCTAATGTTGATGCTGCGTGA 3′ R:5′ GGAGGGGATAATCTTTTGGGAA 3′	60	72
miR-1268b	GSP:5′GAAACGGGCGTGGTGG3′ R:5′GTGCGTGTCGTGGAGTCG3′	60	62
miR-6826-3p	GSP:5′GGCTCCCCTCTCTTTCCT3′ R:5′GTGCGTGTCGTGGAGTCG3′	60	62
SCRG1	F:5′ TCACAACTGTCACAACCTTCCG 3′ R:5′ CATCTCACATCCCTTCCCATCC 3′	60	97
NR4A1	F:5′ GCAAGTGGGCGGAGAAGA 3′ R:5′ CAGGGACATCGACAAGCAAG 3′	60	252
EGLN1	F:5′ GGCAATGGAACGGGTTAT 3′ R:5′ TTGGGTTCAATGTCAGCA 3′	60	164
PLXND1	F:5′ GGCCAAGTAAGCTGCGACAT 3′ R:5′ GAAGTTCCCTACCTGGATTGTG 3′	60	117

SASH1	F:5′ GACGAAGAGCCGCCTTAC 3′<br>R:5′ AACGTGCCGACTTTGTTG 3′	60	179
RUNX1	F:5′ ATGGCACTCTGGTCACTG 3′<br>R:5′ CTGCGGTAGCATTTCTCA 3′	60	72
Beta-actin	F:5′ GTGGCCGAGGACTTTGATTG3′<br>R:5′ CCTGTAACAACGCATCTCATATT3′	60	73
U6	F:5′GCTTCGGCAGCACATATACTAAAAT3′<br>R:5′CGCTTCACGAATTTGCGTGTCAT3′	60	89
GAPDH	F:5′GGGAAACTGTGGCGTGAT3′<br>R:5′GAGTGGGTGTCGCTGTTGA3′	60	299

Notes: cycling conditions were as follows: 95°C for 10 min, followed by 40 cycles of 95°C for 10 s and 60°C for 60 s. Abbreviation: circSCRG1, circular stimulator of chondrogenesis 1; SCRG1, stimulator of chondrogenesis 1; NR4A1, nuclear receptor subfamily 4 group A member 1; EGLN1, egl and nine homologue 1; PLXND1, plexin D1; SASH1, SAM-and SH3-domain containing 1; RUNX1, runt-related transcription factor 1; GAPDH, glyceraldehyde-3-phosphate dehydrogenase.
